# Interprofessional collaboration in diabetes care: perceptions of family physicians practicing in or not in a primary health care team

**DOI:** 10.1186/s12875-019-0932-9

**Published:** 2019-03-13

**Authors:** Olga Szafran, Sandra L. Kennett, Neil R. Bell, Jacqueline M. I. Torti

**Affiliations:** 1 Department of Family Medicine, University of Alberta, 6-10 University Terrace, Edmonton, Alberta T6G 2T4 Canada; 2 Edmonton Oliver Primary Care Network, Family Medicine Clinic, Misericordia Community Hospital, Edmonton, Alberta Canada; 3 Primary Care, Health Canada, Suite 730, 9700 Jasper Avenue, Edmonton, Alberta T5J 4C3 Canada; 4 Department of Family Medicine, University of Alberta, Family Medicine Clinic, Misericordia Community Hospital, 16940 - 87 Avenue, Edmonton, Alberta T5R 4H5 Canada; 5 Department of Family Medicine, University of Alberta, Health Sciences Addition Room 110, London, Ontario N6A 5C1 Canada; 6 Centre for Education Research and Innovation, Schulich School of Medicine and Dentistry, Western University, Health Sciences Addition Room 110, London, Ontario N6A 5C1 Canada

**Keywords:** Type 2 diabetes mellitus, Interprofessional health team, Family physicians, Family practice, Primary health care, Canada

## Abstract

**Background:**

In Canada, most patients with type 2 diabetes mellitus (T2DM) are cared for in the primary care setting in the practices of family physicians. This care is delivered through a variety of practice models ranging from a single practitioner to interprofessional team models of care. This study examined the extent to which family physicians collaborate with other health professionals in the care of patients with T2DM, comparing those who are part of an interprofessional health care team called a Primary Care Network (PCN) to those who are not part of a PCN.

**Methods:**

Family physicians in Alberta, Canada were surveyed to ascertain: which health professionals they refer to or have collaborative arrangements with when caring for T2DM patients; satisfaction and confidence with other professionals’ involvement in diabetes care; and perceived effects of having other professionals involved in diabetes care. Chi-squared and Fishers Exact tests were used to test for differences between PCN and non-PCN physicians.

**Results:**

170 (34%) family physicians responded to the survey, of whom 127 were PCN physicians and 41 were non-PCN physicians (2 not recorded). A significantly greater proportion of PCN physicians vs non-PCN physicians referred patients to pharmacists (23.6% vs 2.6%) or had collaborative working arrangements with diabetes educators (55.3% vs 18.4%), dietitians (54.5% vs 21.1%), or pharmacists (43.1% vs 21.1%), respectively. Regardless of PCN status, family physicians expressed greater satisfaction and confidence in specialists than in other family physicians or health professionals in medication management of patients with T2DM. Physicians who were affiliated with a PCN perceived that interprofessional collaboration enabled them to delegate diabetes education and monitoring and/or adjustment of medications to other health professionals and resulted in improved patient care.

**Conclusions:**

This study sheds new insight on the influence that being part of a primary care team has on physicians’ practice. Specifically, supporting physicians’ access to other health professionals in the primary care setting is perceived to facilitate interprofessional collaboration in the care of patients with T2DM and improve patient care.

## Background

In Canada, most patients with type 2 diabetes mellitus (T2DM) are cared for within the primary care setting [1] and visit their family physicians an average of 8.2 times per year [2]. In response to the increasing prevalence of diabetes, the shortage of endocrinologists, long wait times for referrals to specialists, and the relatively lower cost of managing diabetes in the primary care setting, family physicians are assuming an increasing role in the management of patients with T2DM [3]. Diabetes care within the primary care setting facilitates access for patients and provides more integrated care [4]. This care is delivered through a variety of practice models, ranging from the uni-professional practitioner model to an interprofessional team model of care. Within solo practice, the family physician independently manages the care of diabetic patients and may refer to other practitioners or community programs. In an interprofessional team model of care, different health professionals (family physicians, nurses, diabetes educators, dietitians, pharmacists) work closely together to manage diabetes care, provide services, and refer to community programs, when necessary. Variations exist along the continuum of these two models [1, 5–7], with some physicians working in group practices, but not part of an interprofessional team. Patients with T2DM often present with multiple health conditions and comorbidities which require a chronic disease management perspective adopted by primary health care teams. As such, governments, health care organizations, the World Health Organization, as well as other health care experts, advocate for a collaborative, multifaceted, and multiple strategy approach to dealing with complex health issues and chronic diseases, such as diabetes [8–10].

Interprofessional teams are becoming an integral part of primary health care in Alberta and other provinces within Canada [4]. In Alberta, interprofessional primary health care teams were developed and operate as Primary Care Networks (PCNs) [11] and are comprised of groups of family physicians and other health professionals working together to coordinate the delivery of health services to their patients. One of the key objectives of these interprofessional teams is to support and manage patients with chronic diseases, including T2DM. In many PCNs, diabetes management is supported by a chronic disease management nurse or diabetes educator, pharmacist and/or dietitian working in the practices of family physicians. Generally, interprofessional teamwork has been shown to enhance patient education, improve preventative care, reduce health care costs [12], and result in improved patient outcomes compared to the solo practitioner [13–15]. Interprofessional collaboration has also been shown to result in the reallocation of tasks among team members, allowing physicians more time for other patient care activities [16].

Models of care that employ interprofessional teams in the care of patients with T2DM have been shown to affect processes of care and patient outcomes. The integration of a diabetes education team, consisting of a registered nurse and registered dietitian into primary care practices in Ontario, Canada, was found to increase access to care, improve self-management patient education, improve patient-provider relationships, increase patient satisfaction, and support family physicians in patient care [4]. A study using administrative health data reported that patients with diabetes in practices that were enrolled in a PCN had lower rates of hospital admission and emergency department visits and were more likely to see an ophthalmologist or optometrist and undergo laboratory testing based on recommended guidelines, than patients in a practice not enrolled in a PCN [17]. A team approach to the care of primary care patients with T2DM and depression was also observed to result in improvement in depressive symptoms [18].

Interprofessional collaboration requires the allocation of space, defining team member roles, understanding each professional’s scope of practice, interprofessional interaction, and knowledge exchange between professionals [19]. Within the primary care setting, facilitators of interprofessional collaboration include sharing a common interest in collaboration, opportunities to improve patient care, and the development of new professional skills [20]. Factors influencing collaboration between family physicians and family practice nurses, the largest professional group working in the primary care setting, have included a clear definition of nurses’ roles and responsibilities and trust, respect and communication [21–23]. Hierarchical structures, perceived lack of education and legal liabilities have negatively influenced the ability to collaborate [21–23]. For diabetes care specifically, the increased role of nurses and diabetes educators have been reported to facilitate collaboration, whereas lack of patient motivation and lack of health professionals’ awareness of lifestyle programs were found to hinder collaboration [24]. Patients’ preferences have also been reported to influence collaboration among health professionals [25].

Medication management of patients with T2DM includes components of a comprehensive assessment of medication, medication reconciliation, monitoring, and adjustment, as well as the educating of both patients and practitioners on the safe and efficacious use of medications [26, 27]. Studies indicate that a collaborative pharmacist/physician approach to medication management within primary care settings can improve processes of care and patient outcomes with diabetes [27–29]. The addition of a pharmacist to a primary care team was found to improve blood pressure control through the addition of new medications [30, 31]. A study of diabetes care delivered by an interprofessional team within a family health team setting resulted in improved blood pressure control and TC-HDL levels, an increase in the number of patients being prescribed cardiovascular protective medications, and improved patient understanding of diabetes care [32]. Family practices employing nurse practitioners also observed that diabetic patients with high lipid levels were more likely to receive treatment and increased monitoring of A1C and microalbumin levels [33].

In Alberta, primary health care teams were first developed in 2003 and operate as PCNs [11]. PCNs are comprised of groups of family physicians and other health professionals working together as a team to coordinate the delivery of health services to patients. Each PCN is created through a joint venture agreement between the provincial health authority (Alberta Health Services (AHS)) and a group of family physicians who form a non-profit corporation (NPC) [34]. The physician NPC and AHS jointly govern the PCN and are accountable to the provincial government (Alberta Health) through a grant agreement. The grant provides funding to hire other non-physician health professionals to deliver PCN services to family practices that are affiliated with the PCN. PCN providers report within the PCN management structure. Governance and leadership for PCN planning and coordination are provided by a Provincial PCN Committee and one of five health authority zones. PCN professionals are salaried through the PCN and the physicians generally bill fee-for-service. In the non-PCN setting, physicians generally bill fee-for-service and hire staff and pay office overhead from funds obtained through their billings. In the majority of instances, non-PCN staff provide administrative office services, rather than direct patient care services. In both models of care, physicians consider themselves as being the “most responsible provider” [9] and having the medico-legal responsibility for patient care.

The main goals of PCNs are to increase access to primary care, enhance health promotion and disease prevention, and improve the care of patients with chronic disease and complex health problems. One of the first chronic diseases the PCNs addressed was T2DM. As of September 2013, there were 41 PCNs [35] throughout the province, each consisting of family physicians and other health care providers, such as chronic disease management nurses, pharmacists, dietitians, diabetic educators, and behavioural health consultants. When PCNs were first established, each PCN generally implemented one of two team models – centralized or decentralized. In the centralized model, PCN professionals were located at one central location and patients travelled there to receive services. In the decentralized model, PCN professionals were co-located and distributed within the physicians’ offices and patients received PCN services at their physician’s clinic site. As PCNs matured, some adopted a hybrid model, wherein some services are provided within the family clinic site and other services at the PCN office site. Shared electronic medical records facilitated interprofessional communication and collaboration. While PCNs have been an integral part of primary care reform in Alberta, not all family physicians belong to a PCN; thus, this was an opportune time to examine differences in practice patterns between PCN and non-PCN physicians. Physicians not part of a PCN may also have other health professionals working in the clinic.

Family physicians are a critical part of primary care teams, yet little research has been conducted on how physicians function in interprofessional teams. Being part of an interprofessional team may not necessarily mean that physicians will have collaborative working relationships with other team members. Working relationships within the team are usually established over time and are dependent on the establishment of trust and respect, effective communication, and an understanding of team member roles and responsibilities [31]. The extent of interprofessional collaboration within the PCN setting remains to be examined. As such, the purpose of this study was to examine the extent to which family physicians routinely collaborate with other health professionals in the care of patients with T2DM, comparing those who are part of an interprofessional primary care team (PCN) to those who are not (non-PCN).

## Methods

### Study design, participants & procedures

This was a cross-sectional, anonymous survey of a stratified random sample of 500 family physicians practicing in Alberta. The sampling frame included 2374 family physicians registered with the College of Physicians and Surgeons of Alberta as of January 24, 2013. The list was stratified by urban/rural practice location, with rural being defined as < 10,000 population. The random number generator *Research Randomizer* was used for randomization [36].

A study information letter, the questionnaire, and return pre-paid envelope were mailed to each physician. The physician’s business contact information was obtained from the College of Physicians and Surgeons of Alberta website [37]. A follow-up mail reminder was sent out one month after first initial mail-out. The initial mail-out was conducted during September 2013 and responses were received until January 2014. Consent was implied by the return of a completed questionnaire. The study was approved by Research Ethics Board 2, University of Alberta.

### Questionnaire

A structured questionnaire format with closed-ended questions and multiple response options was employed. The survey questionnaire was designed by the authors and was guided by a combination of the published literature [13, 17, 24], the clinical investigator’s practice experience, and previous research experience. A family physician experienced in providing diabetes care in an interprofessional primary health care team and three health researchers skilled in research methods qualitatively reviewed the questionnaire for face validity. Their comments were incorporated in re-wording, deleting or adding questions that addressed elements of interprofessional collaboration in the care of patients with T2DM.

The outcome measures examined included: physician satisfaction and confidence with other professionals’ involvement in the care of diabetic patients; factors contributing to dissatisfaction; referral to and collaborative arrangements with other health professionals; and physician perceptions of the effect of having other health professionals involved in medication management of diabetic patients. A survey design was selected to facilitate data collection from busy physicians and compare responses from different respondents. It was deemed that more honest responses related to the sensitive topic of physician confidence, satisfaction and dissatisfaction with specific health professionals being involved in the care of patients with T2DM would be obtained in a self-administered survey design than via a qualitative interview or focus group format. While respondents were asked to choose from a list of pre-selected response options, each question also included an “other” option which asked respondents to specify an alternate response.

Neither referral nor collaborative arrangements were explicitly defined in the questionnaire. Referral was assumed to be understood by physicians as the process of directing patients to appropriate health professionals for treatment. Collaborative arrangements were assumed to refer to interactions and sharing of responsibilities for patient care with other health professionals. These assumptions were based on the definition of multidisciplinary team-based care, which was defined in the questionnaire as referring to “a health care team comprised of various health disciplines working collaboratively, with common goals, within a shared setting, to meet the needs of a patient population.” Medication management was defined in the questionnaire as referring to “medication reviews (comprehensive assessment of patient’s medication), medication reconciliation, medication preparation, administration, monitoring, and adjustment, as well as educating of both patients and practitioners on the safe and efficacious use of medications.”

Confidence in other health professionals being involved in the medication management of patients with T2DM was measured on a 3-point scale (1 = Not at all confident, 2 = Somewhat confident, 3 = Very confident). Satisfaction was rated on a 5-point scale (1 = Very dissatisfied, 2 = Somewhat dissatisfied, 3 = Neutral, 4 = Somewhat satisfied, 5 = Very satisfied). Respondents also indicated whether or not their clinical practice was part of a PCN.

### Data analysis

Study data were analyzed using SPSS 24 for Windows. For the purpose of this study, family physicians who indicated that they were part of a PCN (PCN physicians) were deemed to practice in an interprofessional team. Those who were not part of a PCN (non-PCN physicians) were considered to be in a uni-professional practice model, even though they may have been in group partnership with two or more other physicians. During data analysis, *satisfied* was defined as somewhat or very satisfied; *dissatisfied* was defined as very dissatisfied, somewhat dissatisfied or neutral. Sub-group analysis was performed controlling for size of community and clinical practice organization. Chi-squared and Fishers Exact were used to test for differences between PCN and non-PCN physicians. Student’s T-test was used to calculate means. An alpha level of 0.05 was employed to test for statistical significance.

## Results

### Respondents

A total of 170 (34%) family physicians responded to the survey. Of the 168 who indicated their PCN status, 127 (75.6%) were PCN and 41 (24.4%) were non-PCN physicians. Table 1 compares the characteristics of respondents between the two groups. There were no statistically significant differences in mean age (51.2 (range 28–76) vs 49.8 (range 32–83) years), gender (50.4% vs 41.5% female), and average years in clinical practice (20.3 vs 17.5 years) between PCN vs non-PCN physicians. A significantly (*p* = 0.03) greater proportion of PCN physicians practiced in communities of 10,000–200,000 population and non-PCN physicians in metropolitan communities of > 200,000 population. A significantly greater proportion of PCN physicians were organized in a group practice arrangement (*p* = 0.001).

**Table 1:** Characteristics of Respondents

Characteristics	PCN Physicians *n* = 127 (%)	Non-PCN Physicians *n* = 41 (%)
Gender		
Female	64 (50.4)	17 (41.5)
Male	61 (48.0)	24 (58.5)
Not Recorded	2 (1.6)	0 (0.0)
Age Group		
≤ 39 yrs	27 (21.3)	15 (36.6)
40–49 yrs	23 (18.1)	3 (7.3)
50–59 yrs	36 (28.3)	14 (34.1)
≥ 60 yrs	38 (29.9)	9 (22.0)
Not Recorded	3 (2.4)	0 (0.0)
Years in Clinical Practice		
1–10 yrs	39 (30.7)	17 (41.5)
11–20 yrs	20 (15.7)	6 (14.6)
> 20 yrs	65 (51.2)	17 41.5)
Not Recorded	3 (2.4)	1 (2.4)
Size of Community		
Rural (< 10,000 pop)	22 (17.3)	6 (14.6)
Urban (10,000–200,000 pop)	47 (37.0)	7 (17.1)
Metropolitan (> 200,000 pop)	58 (45.7)	28 (68.3)
Clinical Practice Organization		
Solo Practice	16 (12.6)	5 (12.2)
Group Practice	93 (77.5)	17 (51.5)
Interprofessional	11 (8.7)	11 (26.8)
Other	6 (4.7)	8 (19.5)
Not Recorded	1 (0.8)	0 (0.0)

### Referrals & Collaborative Arrangements

**Fig. 1:**
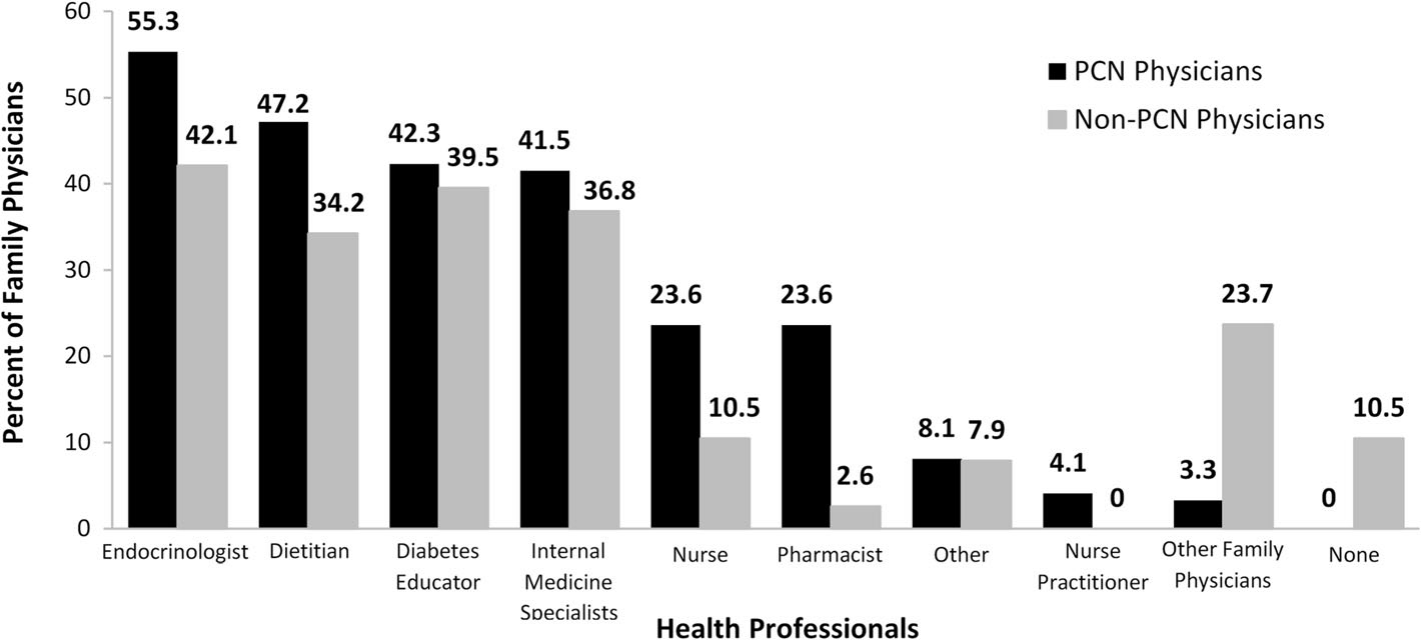
Family Physicians Who Reported Regularly Referring Patients with T2DM to Other Health Professionals

A significantly greater proportion of PCN than non-PCN physicians reported referring patients with T2DM to pharmacists (*p* = 0.003) (Fig. 1), particularly in metropolitan areas (*p* = 0.01). In contrast, more non-PCN physicians did not refer diabetic patients to anyone (p = 0.003) or referred them to other family physicians (*p* < 0.001). Referral to other family physicians was more prevalent among non-PCN than PCN physicians in urban (*p* = 0.04) and metropolitan (*p* = 0.03) communities.

**Fig. 2:**
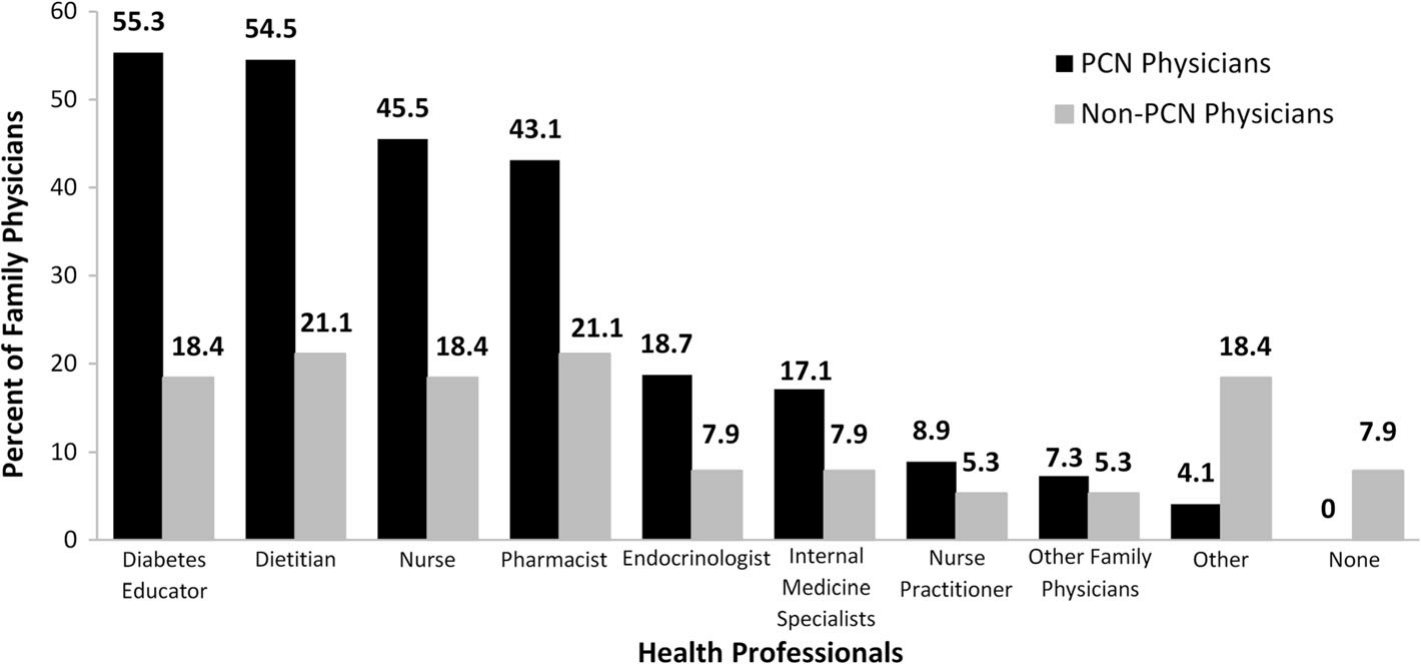
Family Physicians Who Reported Regularly Having Collaborative Arrangements with Other Health Professionals When Caring for Patients with T2DM

Consistently, a significantly greater proportion of PCN physicians reported having regular collaborative working arrangements with diabetes educators (*p* < 0.001), dietitians (*p* < 0.001), nurses (*p* = 0.004), pharmacists (*p* = 0.02), and other health professionals (*p* = 0.01) (Fig. 2). A significantly higher proportion of PCN than non-PCN physicians also reported occasionally referring or having collaborative arrangements with optometrists (74.0% vs 56.4%, *p* = 0.046), nephrologists (71.5% vs 53.8%, *p* = 0.05), cardiologists (60.2% vs 30.8%, *p* = 0.002), home care nurses (43.9% vs 23.1%, *p* = 0.02), and other health professionals (37.4% vs 12.8%, *p* = 0.005). Compared to PCN physicians, a significantly greater proportion of non-PCN physicians did not refer diabetic patients to anyone (4.1% vs 18.6%, *p* = 0.008).

Analysis by size of community revealed that more PCN than non-PCN family physicians in urban areas reported having regular collaborations with nurses (*p* = 0.04), diabetes educators (*p* = 0.02), dietitians (*p* = 0.04), and pharmacists (*p* = 0.04). Similarly, in metropolitan areas a significantly greater proportion of PCN physicians indicated having regular collaborative working arrangements with nurses (*p* = 0.03), diabetes educators (*p* = 0.003), and dietitians (*p* = 0.004). Whereas more PCN than non-PCN physicians in metropolitan areas occasionally referred or had collaborative arrangements with cardiologists (*p* = 0.03) or other health professionals (*p* = 0.001), significantly more PCN physicians in rural (p = 0.001) and urban (*p* = 0.04) communities referred to home care nurses.

Analysis by practice organization showed that a significantly greater proportion of PCN family physicians in group practice had regular collaborations with nurses (*p* = 0.01), diabetes educators (*p* = 0,03), and dietitians (*p* = 0.01).

### Confidence & satisfaction

There were no statistically significant differences between PCN and non-PCN physicians in their confidence with other health professionals being involved in the medication management of patients with T2DM. Collectively, family physicians were very confident in endocrinologists (94.0%), internal medicine specialists (88.8%), and other family physicians (58.2%). With respect to other health professionals, physicians indicated they were very confident in diabetes educators (65.2%) and pharmacists (63.8%), but less so in nurse practitioners (44.4%), dietitians (43.2%), and nurses (41.7%) with respect to medication management.

No statistically significant differences were noted between PCN and non-PCN physicians in their satisfaction with other health professionals being involved in the medication management of patients with T2DM. Overall, more family physicians were satisfied with specialists (endocrinologists 94.0%, internal medicine specialists 90.2%) than with other family physicians (69.4%). Satisfaction with other health professionals was: pharmacists 85.9%; diabetes educators 84.0%; nurses 73.7%; dietitians 72.1%; nurse practitioners 67.6%.

There were also no significant differences between PCN and non-PCN physicians in factors contributing to dissatisfaction with other health professionals being involved in medication management of patients with T2DM. Overall, these factors included: lack of training and/or medical knowledge (36.3%); lack of collaboration due to space and time (32.2%); lack of information technology to allow for information sharing (28.8%); inability to supervise staff (19.9%); lack of trust (13.0%); and lack of resources (staff, equipment) (11.0%). Of the total respondents, 28.1% reported no factors contributing to dissatisfaction.

### Perceived Outcomes & Benefits

**Fig. 3:**
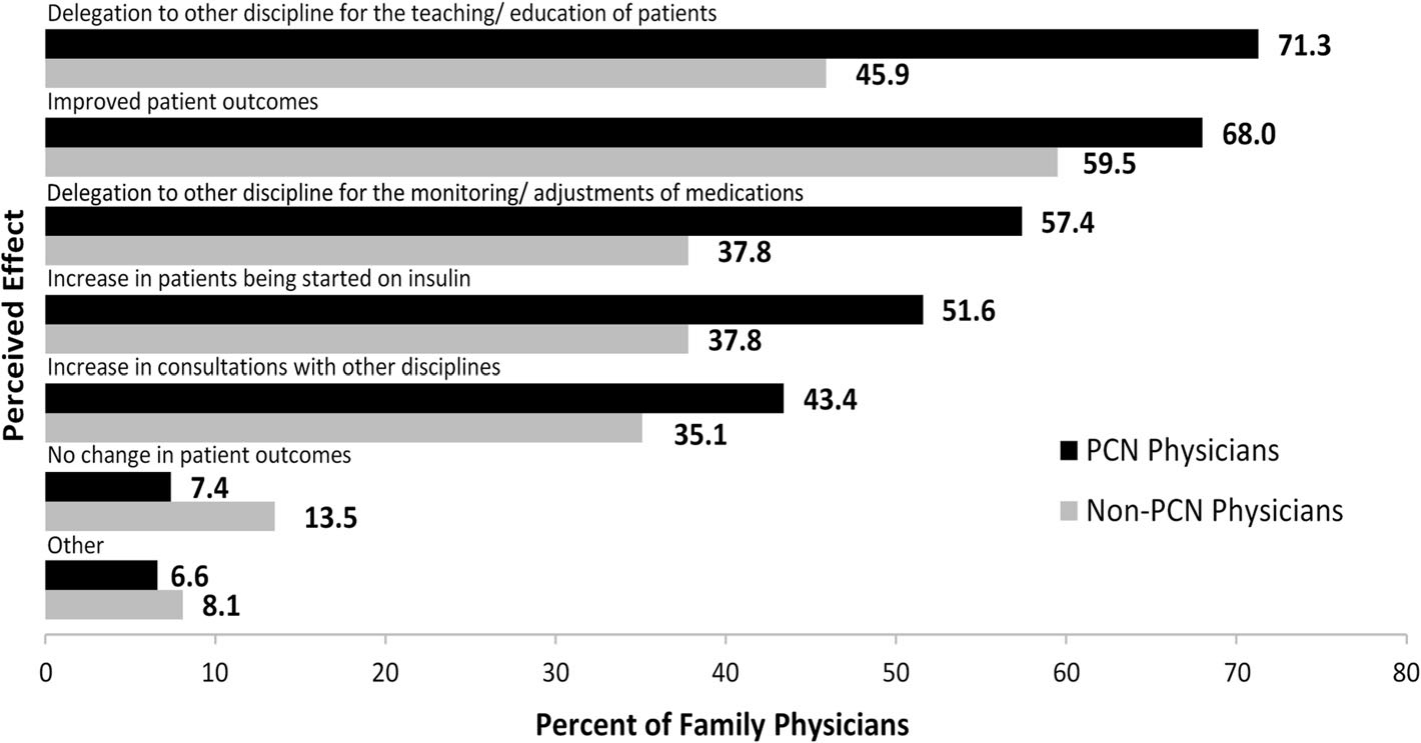
Perceived Effects of Having Other Health Professionals Involved in Medication Management of Patients with T2DM

Overall, the perceived effects of having other health professionals involved in medication management of patients with T2DM included: improved patient outcomes (66.0%); delegation of patient teaching (65.4%); delegation to other disciplines of the monitoring and adjustment of diabetes medications (52.8%); an increase in patients being started on insulin (48.4%); and an increase in consultations regarding diabetes medications with other disciplines (41.5%). Only 8.8% of physicians indicated that they perceived no changes in patient outcomes in having other health professionals involved in the medication management of patients with T2DM. A significantly greater proportion of PCN than non-PCN physicians were of the opinion that team-based care of T2DM patients resulted in the delegation of patient teaching (*p* = 0.006) and of the monitoring and adjustment of diabetes medications (*p* = 0.04) to other disciplines (Fig. 3); both were significantly more prevalent among PCN physicians in metropolitan area (*p* = 0.02).

## Discussion

The study findings reveal that family physicians who are affiliated with a PCN report involving other health professionals in the care of patients with T2DM to a greater degree than family physicians who are not part of a PCN. This is evidence that supporting physicians’ access to other health professionals in the primary care setting facilitates interprofessional collaboration. Although interprofessional collaboration was comparatively greater among family physicians who worked as part of a team than those who did not, in absolute terms the degree of collaboration did not appear to be very high. Many factors contribute to the extent to which health care professionals work together including the development of trust, effective communication, and clear role definitions [38]. Some combination of these factors (facilitators and barriers) may account for the reported percentage of interprofessional collaboration not being higher. While the findings of this study do not reveal the nature of the interprofessional working relationship, published research [39, 40] indicates that family physicians perceive themselves to have the leadership role in the health care team. Lower levels of collaboration may also be attributed to some family physicians providing focused clinical care (e.g. sports medicine), rather than comprehensive practice, and thus not having many patients with T2DM in their practice. While there is opportunity to increase interprofessional team collaboration in the care of patients with T2DM, limiting factors may include the availability of other health professionals and a willingness to overcome traditional professional roles.

Family physicians perceive that interprofessional teamwork enables them to delegate patient education to nurses and diabetes educators and the monitoring and adjustment of diabetic medications to pharmacists. The delegation of tasks was also identified in a study of family physicians’ perspectives on interprofessional teamwork [40]. This reflects a growing recognition by family physicians that other health professionals have more appropriate knowledge and expertise and often more time than physicians to perform these roles. This is encouraging as diabetes is a complex chronic condition that requires a multifaceted interprofessional and patient-mediated approach to management.

The finding that significantly more family physicians who were not part of a PCN did not refer patients with T2DM to anyone may reflect the provision of episodic care or having patients whose diabetes is well-controlled. Patients with T2DM who receive episodic care may have unmet needs and may not receive adequate follow-up or not be well-controlled [41, 42]. Those physicians who referred to other family physicians may be practicing shared responsibility in the care of diabetic patients with family physician colleagues, particularly within larger group practices, wherein some physicians may develop special interests in managing particular conditions and acquire a referral base from their family practice colleagues. It is also possible that non-PCN physicians may refer their patients to other PCN family physicians to gain access to PCN services.

Family physicians indicated having highest confidence and satisfaction in specialists and lower confidence in other health professionals with medication management of patients with T2DM. A similar pattern was observed in terms of satisfaction with other health professionals being involved in medication management. Of note is that the highest levels of confidence and satisfaction were attributed to specialists to whom family physicians refer patients to, but with whom they do not work with directly. Family physicians may be ascribing high expressions of confidence and satisfaction to a perception of the ability to manage the disease, high education levels, and perceived medical skills of specialists, rather than formulating impressions based on direct encounters with these health professionals. This may reflect the traditional relationships between family physicians and specialists in the management of patient care. For family physicians, it may be easier to trust the traditional system that they are familiar with, rather than a new system of interprofessional care wherein they are unsure of the skills of the other team members. The pattern of these findings may be seemingly indicative of a hierarchy among the health care professions. The comparatively higher levels of reported satisfaction than of confidence may reflect the notion that confidence implies some assertion of certainty, whereas satisfaction is more of a subjective phenomenon. These findings suggest that moving family physicians toward a culture of interprofessional teamwork in diabetes care requires overcoming traditional professional roles and establishing professional trust and confidence.

Despite differences in referral rates to pharmacists, there were no differences in family physicians’ confidence with other health professionals being involved in medication management of patients with T2DM. Differences in referral rates can be attributed to differences in access, i.e. physicians who are part of PCNs have greater access to pharmacists, thus are more likely to refer to them. Confidence ratings are more likely based on perceptions of skill and knowledge of health professionals and these perceptions are less likely to be associated with PCN status of physicians.

This study was limited in that it was a cross-sectional survey and data were collected only at one point in time; as such, the evolution of interprofessional team collaboration was not captured. Classification of physicians working in interprofessional teams was based on whether or not they were part of a PCN. Being affiliated with a PCN may not directly translate into physicians being active members of an interprofessional team and having collaborative working relationships with other team members. Conversely, not being part of a PCN may not necessarily mean that a family physician independently cares for patients with T2DM. It was not possible to determine from the questionnaires if any of the physicians were in the same practice, as the survey was anonymous and did not ask respondents to identify which PCN they were affiliated with. As such, it was not possible to explore cluster effects, nor analyze differences in response rate by PCN. Physician participation in PCNs is voluntary, thus greater interprofessional collaboration of PCN physicians in the care of patients with T2DM may be attributed in part to self-selection bias, with those physicians who are more inclined to work in teams being more inclined to join PCNs*.* The analysis was based on self-reported measures and is subject to bias. The findings on the perceived outcomes and benefits of interprofessional collaboration are based on subjective assessments of perceived changes and not on quantifiable measures. A before-after study design is needed to provide more rigorous data. Nevertheless, physicians’ perceptions about outcomes of collaborative diabetes care can provide valuable insight into their perceptions of interprofessional collaboration and can be a valuable supplement to quantifiable data obtained from more rigorous evaluation. Referral and collaborative arrangements were not defined in the questionnaire but assumed to be understood by practicing physicians. Given that family physicians refer patients to various health providers on a daily basis, there should not be wide variation in the interpretation of referral by the respondents. While providing care, physicians also collaborate and interact with other providers on a regular basis, the degree to which they “share” responsibility for patient care with other health professionals is unknown and may vary between respondents, resulting in variability in the interpretation of collaborative arrangements. The findings of the sub-group analysis should be interpreted with caution given that in some instances the frequencies were relatively low. As such, the influence of size of community and practice organization on PCN vs non-PCN physicians remains to be elucidated. We speculate that larger communities would have more availability of resources and the presence of a variety of health professionals, thus more options for collaboration by PCN physicians. Similarly, those in group or interprofessional practice may be more likely to join a PCN as they may be already predisposed to collaboration in their practice organization. The study is somewhat limited by its relatively low response rate of 34%. Based on the study population size of 500 and a 95% confidence level, the 170 respondents resulted in a slightly higher 6.1% rather than the usual 5% margin of error. As such, there may be potential selection and/or response bias. While the response rate was on the lower end, it is reasonable for a postal survey of physicians. A systematic review of Portuguese primary care physicians’ response rate to surveys reported substantial heterogeneity, with the average response rate to postal surveys being 37% and larger studies (≥ 500 participants) having lower response rates [43]. The closed-ended questionnaire format may have been limited in providing somewhat simplistic responses to complex issues. A supplementary qualitative component to the study would have facilitated a more in-depth understanding of the issues.

Future studies should examine objective measures of family physician referrals to and collaborative arrangements with other health professionals in the care of patients with T2DM. In addition, an examination of facilitators and barriers to interprofessional teamwork in the care of patients with T2DM in the primary care setting is warranted.

## Conclusions

This study provides new and relevant information on family physicians’ perceptions of interprofessional collaboration in the care of patients with T2DM and on the influence that being part of a primary care team has on physicians’ practice. Family physicians who are part of a PCN report involving other health professionals in the care of T2DM patients to a greater extent than those who are not affiliated with a PCN. Family physicians perceive the effects of interprofessional teamwork in the care of patients with T2DM to be the delegation to other disciplines for teaching/education and monitoring and adjustment of diabetic medications, as well as improved patient care. While there is opportunity to increase interprofessional collaboration in the care patients with T2DM within PCNs, limiting factors may include the availability of other health care professionals and a willingness to overcome traditional professional roles. The findings add to the knowledge-base and support the importance of pursuing research and implementation efforts to change and improve practice.

## Abbreviations

T2DM: Type 2 diabetes mellitus; PCN: Primary Care Network
